# An infrapatellar nerve block reduces knee pain in patients with chronic anterior knee pain after tibial nailing: a randomized, placebo-controlled trial in 34 patients

**DOI:** 10.1080/17453674.2019.1613808

**Published:** 2019-05-09

**Authors:** Mandala S Leliveld, Saskia J M Kamphuis, Michael H J Verhofstad

**Affiliations:** a Trauma Research Unit, Department of Surgery, Erasmus MC, University Medical Center Rotterdam, Rotterdam;; b Department of Surgery, Gelderse Vallei Hospital, Ede, The Netherlands

## Abstract

Background and purpose — Anterior knee pain is common after tibial nailing. Its origin is poorly understood. Injury of the infrapatellar nerve is a possible cause. In this randomized controlled trial we compared changes in knee pain after an infrapatellar nerve block with lidocaine or placebo in patients with persistent knee pain after tibial nailing.

Patients and methods — Patients with chronic knee pain after tibial nailing were randomized to an infrapatellar nerve block with 5 ml 2% lidocaine or placebo (sodium chloride 0.9%), after which they performed 8 daily activities. Before and after these activities, pain was recorded using a numeric rating scale (NRS; 0–10). Primary endpoint was the change in pain during kneeling after the infrapatellar nerve block. Secondary outcomes were changes in pain after the nerve block during the other activities.

Results — 34 patients (age 18–62 years) were equally randomized. A significant reduction of the NRS for kneeling pain with an infrapatellar nerve block with lidocaine was found compared with placebo (–4.5 [range –10 to –1] versus –1 [–9 to 2]; p = 0.002). There were no differences between the treatments for the NRS values for pain during other activities.

Interpretation — Compared with placebo, an infrapatellar nerve block with lidocaine was more effective in reducing pain during kneeling in patients with chronic knee pain after tibial nailing. Our findings support the contention that kneeling pain after tibial nailing is a peripheral nerve-related problem.

The common treatment for tibial shaft fractures is intramedullary nailing. A drawback of this procedure is anterior knee pain (Katsoulis et al. 2006). Persisting knee pain after more than 8 years post-nailing is reported with restrictions in daily and leisure activities (Lefaivre et al. 2008, Leliveld and Verhofstad 2012, Larsen et al. 2014). Removal of the nail does not alleviate pain in all patients and can even initiate anterior knee pain in some (Boerger et al. 1999). The cause of this phenomenon is unknown. Among the structures at risk for injury during tibial nailing through an infrapatellar approach is the infrapatellar branch of the saphenous nerve. Injury to this nerve usually results in numbness on the anterior aspect of the knee and the proximal lateral part of the lower leg. This complication has been reported after several other surgical procedures around the knee, such as knee arthroscopy (Mochida and Kikuchi 1995) and anterior cruciate ligament reconstruction (Spicer et al. 2000). In addition, development of post-procedural neuropathic pain has been described (Dellon et al. 1996). Since the infrapatellar nerve runs perpendicular to the patellar tendon, the nerve is at risk for transection during tibial nailing (Mochida and Kikuchi 1995, Kerver et al. 2013). Injury to the infrapatellar nerve after tibial nailing has been reported (Lefaivre et al. 2008, Leliveld and Verhofstad 2012), and sensory deficits of the infrapatellar nerve have been associated with chronic anterior knee pain after tibial nailing (Leliveld and Verhofstad 2012). However, studies to examine a causative relation between infrapatellar nerve injury and anterior knee pain after tibial nailing have not yet been conducted.

We hypothesized that if knee pain after tibial nailing is indeed caused by neuropathic pain due to injury or entrapment of the infrapatellar nerve, an anesthetic block of this nerve with lidocaine will reduce knee pain in these patients.

## Patients and methods

### Patients

From the medical record systems and charting database patients between 18 and 65 years old, treated with an intramedullary nail for an isolated traumatic unilateral tibial shaft fracture (AO/OTA 42 A–C) between June 2000 and December 2016, were selected from St Elisabeth Hospital (Tilburg, The Netherlands, level 1 trauma center and teaching hospital), Gelderse Vallei Hospital (Ede, The Netherlands, level 2 trauma center and teaching hospital), and Erasmus Medical Center (University Medical Center Rotterdam, The Netherlands, level 1 trauma center and teaching hospital). Nailing or nail removal had to be more than 6 months ago. 601 patients with a tibial shaft fracture were treated with an intramedullary nail introduced through a longitudinal infrapatellar incision during the specified period (Figure). After application of the exclusion criteria, 407 patients were potentially eligible for trial participation. These patients were sent a numeric rating scale (NRS) to rate knee anterior pain during 8 different daily activities (kneeling, squatting, prolonged sitting with bent knees, jumping, walking on stairs, running, walking, and rest). If the patient did not reply, telephone calls were attempted. Pain scores were returned by 233 patients. Criteria for inclusion in the trial were an NRS of 4–6 (moderate pain) during at least 3 out of 8 activities or an NRS of 7 or higher (severe pain) during 1 or more activities. 79 patients met these criteria, of whom 34 agreed to participate in the study.

### Study design and assessment

Eligible patients who agreed to participate in this study were seen at the outpatient clinics of the participating hospitals. Trauma characteristics, data concerning the initial procedure and nail removal were gathered retrospectively. Length of the longitudinal incision was measured on a flexed knee in mm using a tape measure and localization of the incision was noted (on patellar tendon or medial to patellar tendon). Sensory disturbances (numbness, hypesthesia, allodynia) in the area of the infrapatellar nerve (anterior and lateral aspect of the knee) were tested using a cotton swab, comparing the non-operated leg with the operated leg and the surrounding dermatomas. Baseline pain (T0) was scored using an NRS during 8 activities, which were all supervised by an examiner (SJMK or MSL). The NRS measures pain severity by asking the patient to select a number (from 0 to 10) to represent how severe the pain is, where 0 represents “no pain” and 10 represents “worst pain possible.” This rating scale has shown to be valid, reliable, and appropriate for use in clinical practice (Williamson and Hoggart 2005, Hjermstad et al. 2011), is responsive in patients with chronic nociceptive or neurogenic pain (Lundeberg et al. 2001), and the minimally clinical important change has been determined in patients with chronic musculoskeletal pain (Farrar et al. 2001, Salaffi et al. 2004). If a patient was not able or willing to perform an activity it was noted as a missing value.

Equal randomization of the treatment sequence was performed with use of a random-number generator. The allocated sequence was kept in sealed envelopes. Randomization and preparation of the envelopes by a secretary who had no involvement in the trial. Upon each patient’s enrollment into the study, the next consecutively numbered envelope was opened by an outpatient nurse. Lidocaine 2% and sodium chloride 0.9% (saline) were used for the nerve blocks. 2 syringes were prepared, marked with number 1 or 2 according to the allocation, and checked by a doctor not involved in the trial. The name and date of birth of the participant were written on the envelope. As both fluids were colorless and odorless, both patient and examiner remained unaware of which treatment was administered.

An infrapatellar nerve block was performed freehand by depositing 5 mL of the solution in a fan-like manner subcutaneously between the medial surface of the medial femoral condyle and the medial aspect of the patellar tendon, including the incision. After 5 minutes patients completed the 8 activities under supervision and NRS scores were subsequently obtained (T1). Thereafter, each patient crossed over and was injected with the alternate treatment (T2). Time between injections (wash-out period) was approximately 30 minutes.

Kneeling is the most frequently and painful activity reported after tibial nailing (Court-Brown et al. 1997, Toivanen et al. 2002, Cartwright-Terry et al. 2007, Vaisto et al. 2008). Therefore, the primary endpoint was the change in pain intensity during kneeling after infrapatellar nerve block with lidocaine and placebo, measured using an NRS. Secondary outcomes were changes in pain intensity after each nerve block as measured using an NRS during the 8 activities.

### Sample-size calculation

A mean NRS of 7 for kneeling pain in patients with chronic anterior knee pain after tibial nailing was used for sample-size calculation (Salaffi et al. 2004). A change in pain intensity of > 30% was considered clinically meaningful (Farrar et al. 2001, Salaffi et al. 2004). Using a 2-sided test, an α level of 0.05, and a power of 80%, 34 patients were needed to be enrolled.

### Statistics

Normally distributed continuous data are presented as mean (SD). Skewed data are presented as median (range). Differences between the 2 groups were tested using Student’s t-test (normal distribution) or Mann–Whitney U test (skewed distribution) for continuous variables. The method described by Hills and Armitage for two-period cross-over clinical trials was applied (Hills and Armitage 2004). Treatment effect was calculated with (T1–T0) – (T2–T1) for the lidocaine–placebo sequence group and (T2–T1) – (T1–T0) for the placebo–lidocaine sequence group. Period effect (the response to a treatment during the second period is not influenced by the treatment which was given during the first period) was calculated with (T1–T0) – (T2–T1) for both groups. Both treatment effect and period effect were analyzed using Mann–Whitney U test (Hills and Armitage 2004). In cases where a period effect was present, the results from the first nerve block only were analyzed. p < 0.05 was considered statistically significant. Data analysis was performed using SPSS version 25.0 for Windows (IBM Corp, Armonk, NY, USA).

### Ethics, registration, funding, and potential conflicts of interest

Approval was obtained from the central medical research ethics committee and the institutional board of all participating hospitals (NL34510.008.11/P1142 2016/07/20). The study was registered with the Dutch trial registry (NTR4628; Nederlands Trial Register; http://www.trialregister.nl). Written informed consent was obtained from all patients. Participants did not receive compensation of any kind. The study was not funded by any source. The authors have no competing interests to declare.

## Results

### Patient characteristics

Baseline demographics, length of the incision, placement of the incision, and sensory disturbances of the infrapatellar nerve are displayed in Table 1. Median age was 46 years (18–62). Median follow-up was 86 months (6–168). 85% of the patients showed signs of injury to the infrapatellar nerve (numbness, hypesthesia, or allodynia).

**Table 1.** Baseline characteristics

Factor	Lidocaine group (n = 17)	Placebo group (n = 17)
Male sex	9	10
Age, median (range)	40 (22–62)	38 (18–60)
AO/OTA fracture classification type		
A	7	8
B	9	7
C	1	2
Months after tibial nailing, median (range)	80 (6–168)	67 (11–168)
Tibia nail removed	9	12
Length of longitudinal infrapatellar		
incision (mm), mean (SD)	58 (17)	58 (12)
Placement of incision		
medial to patellar tendon	2	2
midbundle of patellar tendon	15	15
Sensory disturbance infrapatellar nerve	15	14

### Pain scores

All patients received the infrapatellar nerve block according to group allocation. Pain scores at baseline (T0), after the first nerve block (T1), and after the second nerve block (T2) are displayed in Table 2. Kneeling was the most painful activity, followed by squatting. Some participants were not able or willing to perform all 8 activities.

**Table 2.** Change in pain scores after nerve block with lidocaine and placebo. Values are presented as median (range)

Activity	n^a^	Lidocaine	Placebo	n^a^	Placebo	Lidocaine	Treatment effect^b^	Period effect^b^
Kneeling	14	–4.5 (–10 to –1)	0 (–4 to 1)	16	–1 (–9 to 2)	–1.5 (–8 to 2)	0.02	0.004
Squatting	14	–3 (–9 to 1)	0 (–4 to 9)	15	–1 (–9 to 1)	0 (–7 to 2)	0.03	0.09
Sitting with bent knees	15	–3 (–7 to 0)	0 (–1 to 1)	17	–2 (–6 to 0)	0 (–7 to 9)	0.001	1.0
Jumping	11	–1 (–6 to 2)	0 (–1 to 1)	12	–1 (–3 to 1)	–1 (–3 to 1)	0.09	0.3
Walking on stairs	17	0 (–6 to 2)	0 (–1 to 1)	17	0 (–5 to 2)	0 (–4 to 3)	0.4	0.4
Running	11	0 (–5 to 2)	0 (–3 to 8)	11	0 (–3 to 1)	0 (–5 to 0)	0.6	0.8
Rest	17	0 (–1 to 1)	0 (–1 to 1)	17	0 (–1 to 6)	0 (–3 to 1)	0.2	0.1
Walking	17	0 (–5 to 1)	0 (–1 to 2)	17	0 (–4 to 4)	0 (–5 to 3)	0.5	0.4

a Not all patients performed all activities.

b Mann–Whitney U.

Treatment effects (decline in median pain scores) were significant for kneeling (p = 0.02), squatting (p = 0.03) and sitting with bent knees (p = 0.001). However, a period effect was present for the primary endpoint kneeling (Table 2), meaning the intervention exerted a different effect in the first period (T1–T0) than in the second period (T2–T1). We therefore chose to additionally analyze the results from the first nerve block (T1–T0), like a randomized trial comparing 2 groups.

For kneeling a significant decline in median pain scores remained after a nerve block with lidocaine compared with placebo (–4.5 [–10 to –1] versus –1 [–9 to 2]; p = 0.002). There was no statistically difference between the groups during squatting, sitting with bent knees, jumping, walking stairs, running, walking, and rest (data not shown).

## Discussion

The purpose of this study was to compare changes in knee pain after a subcutaneous lidocaine block of the infrapatellar nerve or placebo in patients with chronic anterior knee pain after tibial nailing. For kneeling a significant reduction in pain scores was found after an infrapatellar nerve block with lidocaine.

The effect of lidocaine usually lasts about 1–2 hours and with a wash-out period of about 30 minutes one can presume that the effect of the lidocaine injection persists during the second treatment period. We expected that pain scores in the lidocaine-first group would reach their utmost lowest levels after injection and only minimal changes would occur after the second injection with saline. Pain scores in the placebo-first group were expected to decline only minimally after the first injection and decline further after lidocaine injection; a difference in change scores would then still be observed. However, data analysis showed a period effect for the primary endpoint, meaning the effect of the treatment was different in the first period (T1–T0) from the effect in the second period (T2–T1). This can easily be explained by the short wash-out period. Also, both the patient and the examiner were blinded to the treatment given. Due to the local effect lidocaine has on the skin, patients may recognize this effect. This affects true blinding and might also have affected the pain scores.

Although pain during kneeling was reduced in both groups, pain was not totally diminished and no statistically significant reduction was seen for pain during the other activities (squatting, sitting with bent knees, jumping, running, walking on stairs, walking, and at rest). A possible explanation is that not all patients were able or willing to do these activities, which affected the statistical power. Moreover, the starting pain level was lower than in other activities, thus a smaller effect size can be expected. The study could be underpowered for these activities; however, they were not the primary outcome. In some patients pain can be multi-modal and might as well have originated from intra-articular injury (Hernigou and Cohen 2000) or irritation of Hoffa’s fat pad (Jankovic et al. 2013).

Knee pain is a common complaint after intramedullary nailing for tibial shaft fractures. In this study 79 of 233 patients (34%) who returned their NRS indicated they had either moderate or severe knee pain during several activities after a median follow-up of 7.1 years (0.5–14). Although there might be some selection bias due to selective response to the initial questionnaire, this percentage is in concordance with the long-term results of Lefaivre et al (2008) and Leliveld and Verhofstad (2012), who respectively reported 29% and 38% of chronic knee pain after tibial nailing after a median follow-up of 14 and 7 years.

Kneeling pain is frequently mentioned to be the most painful activity (Court-Brown et al. 1997, Toivanen et al. 2002, Cartwright-Terry et al. 2007, Vaisto et al. 2008). In a randomized trial comparing 2 different incisions from Toivanen et al. (2002), 62% of the patients stated kneeling pain as being most painful. The mean visual analogue score (0–100 mm) for kneeling pain in these patients was 31 mm (transtendinous approach) and 44 mm (paratendinous approach). In a retrospective study Court-Brown et al. (1997) even reported kneeling pain as the most painful activity in 92% of their patients, followed by squatting (61%). The average scores for these activities on a 10-point analogue scale were respectively 3.1 and 3.3. The fact that kneeling pain scores in our study are higher (median NRS of 8.0) is due to the fact that patients were selected based on their pain scores (scores of 4 or higher for at least 3 activities or 7 and higher for at least one activity).

We detected sensory disturbances in the area of the infrapatellar nerve (anterior and lateral aspect of the knee) in 29/34 of the patients. Iatrogenic injury to the infrapatellar nerve is one of many concepts regarding the origin of anterior knee pain after tibial nailing. The course of the infrapatellar nerve makes it susceptible to iatrogenic injury during nail insertion, especially when longitudinal infrapatellar medial and midline incisions are used (Kerver et al. 2013). Long-lasting sensory deficits at the anterior aspect of the knee are described after tibial nailing (Karladani et al. 2007, Lefaivre et al. 2008, Leliveld and Verhofstad 2012) and a correlation was found with anterior knee pain after tibial nailing (Leliveld and Verhofstad 2012). Nahabedian and Johnson (2001) performed a selective infrapatellar nerve denervation in 9 patients with chronic knee pain after blunt trauma to the knee and total knee replacement. Median pain scores (NRS 0–10) reduced from 8.0 (range 7–10) at baseline to 3.0 (range 0–6) after the denervation and they conclude that selective denervation is a beneficial procedure in selected patients with neuromatous knee pain. An infrapatellar nerve block with lidocaine in our study showed a significant difference in change of pain intensity during kneeling in patients treated with an intramedullary nail, compared with a nerve block with placebo. Because the infrapatellar nerve solely provides sensation of the skin at the antero-lateral aspect of the knee, an anesthetic block with lidocaine can diminish cutaneous neuropathic pain in this region (Nahabedian and Johnson 2001, Hsu et al. 2013) but not pain related to intraarticular injury.

Although pain scores declined for all activities at the end of the study, actual function and effect on function were not assessed. Sudden improvement of functional outcome was, however, not expected in patients who sustained knee pain for several years. Long-term improvement in function after infrapatellar nerve block has been reported though (Hsu et al. 2013), as has long-term pain relief after denervation of the infrapatellar nerve (Dellon et al. 1996, Nahabedian and ­Johnson 2001).

The incidence of persisting anterior knee pain after tibial nailing is high and we provide arguments for the hypothesis that iatrogenic injury to the infrapatellar nerve contributes to this problem. Patients suffering from this complication who response well to an infrapatellar nerve block with lidocaine might benefit from denervation (Dellon et al. 1996, Nahabedian et al. 1998, Nahabedian and Johnson 2001). Based on anatomical studies a transverse or oblique incision would yield the least chance of injury to or entrapment of the infrapatellar nerve (Mochida and Kikuchi 1995, Ebraheim and Mekhail 1997, Kerver et al. 2013). Alternatively, the suprapatellar approach for tibial nailing avoids the risk zone for infrapatellar nerve injury (Kerver et al. 2013) and studies have reported low knee pain scores and good functional results after this approach (Chan et al. 2016, Sun et al. 2016, Rothberg et al. 2019).

In summary, compared with placebo, an infrapatellar nerve block with lidocaine was more effective in reducing pain during kneeling in patients with chronic knee pain after tibial nailing through a longitudinal infrapatellar incision. Our data support the contention that kneeling pain after tibial nailing is a peripheral nerve-related problem.

**Figure:**
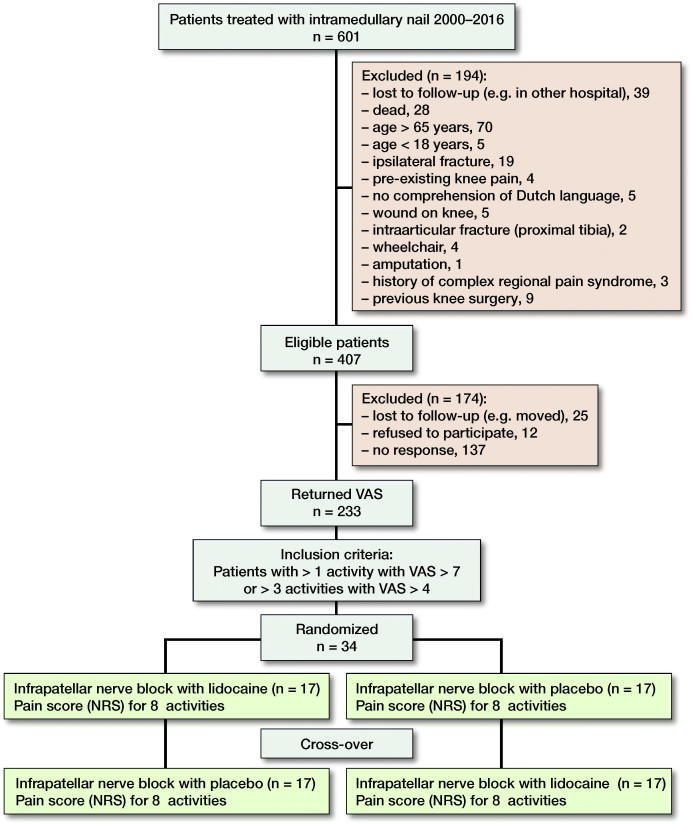
Patient selection, allocation and study design.
